# The menopause-obesity axis in MASH progression: from estrogen decline to liver fibrosis

**DOI:** 10.3389/fgstr.2026.1889458

**Published:** 2026-08-13

**Authors:** Anastasia Ntikoudi, Eugenia Vlachou

**Affiliations:** Department of Nursing, University of West Attica, Athens, Greece

**Keywords:** liver fibrosis, MASH, MASLD, menopause, obesity

## Abstract

Metabolic dysfunction-associated steatotic liver disease (MASLD) and metabolic dysfunction-associated steatohepatitis (MASH) have emerged as major global health challenges, particularly among individuals with obesity and metabolic dysfunction. Menopause represents a critical hormonal transition characterized by estrogen deficiency, visceral adiposity, insulin resistance, and chronic low-grade inflammation, all of which may accelerate MASH progression and hepatic fibrosis in women. This narrative review summarizes current evidence regarding the menopause-obesity axis in MASH progression, focusing on molecular mechanisms, histopathological alterations, diagnostic biomarkers, imaging modalities, and emerging therapeutic strategies. Estrogen deficiency contributes to metabolic dysregulation through impaired insulin signaling, increased visceral adiposity, lipotoxicity, oxidative stress, and adipose tissue inflammation, thereby promoting hepatic steatosis and progression toward fibrosis. Histopathological progression is characterized by steatosis, hepatocyte ballooning, chronic inflammation, and extracellular matrix deposition. Non-invasive diagnostic approaches, including serum biomarkers, transient elastography, controlled attenuation parameter (CAP), and magnetic resonance imaging proton density fat fraction (MRI-PDFF), have improved disease detection and risk stratification. Lifestyle modification and sustained weight reduction remain the cornerstone of management, while emerging pharmacologic therapies such as glucagon-like peptide-1 receptor agonists, tirzepatide, and resmetirom demonstrate promising metabolic and hepatic benefits. Hormone-based and anti-fibrotic therapies may offer additional therapeutic potential in postmenopausal women with obesity-associated MASH. Collectively, the menopause-obesity axis appears to play a central role in hepatic injury and fibrosis progression through interconnected hormonal, metabolic, and inflammatory pathways. Improved understanding of sex-specific mechanisms may facilitate the development of personalized diagnostic and therapeutic approaches for postmenopausal women with MASLD and MASH.

## 1 Introduction

Metabolic dysfunction-associated steatotic liver disease (MASLD) has emerged as the most common chronic liver disease worldwide and currently affects approximately 30% of the global population (1, 2). The disease spectrum ranges from simple hepatic steatosis to metabolic dysfunction-associated steatohepatitis (MASH), progressive fibrosis, cirrhosis, and hepatocellular carcinoma, contributing substantially to liver-related and all-cause mortality (3). Epidemiological studies have demonstrated a steady rise in MASLD-associated morbidity and mortality over recent decades, particularly in parallel with the increasing prevalence of obesity, type 2 diabetes mellitus, and metabolic syndrome (4, 5). Pathophysiologically, the progression of MASLD is closely linked to insulin resistance, chronic low-grade inflammation, oxidative stress, and gut microbiota dysregulation, all of which promote hepatic injury and fibrogenesis (6). Moreover, delayed diagnosis, limited public awareness, and restricted access to effective pharmacological therapies continue to contribute to adverse clinical outcomes in affected populations.

Age is an important determinant of disease progression and liver-related mortality in MASLD/MASH. Older individuals exhibit a significantly higher risk of advanced fibrosis, cirrhosis, and hepatocellular carcinoma compared with younger populations (7, 8). In addition, substantial ethnic and racial disparities have been reported in disease prevalence, severity, and clinical outcomes, potentially reflecting differences in genetic susceptibility, lifestyle factors, socioeconomic status, and metabolic health profiles (9–11). Sex-related differences have also become increasingly recognized in MASLD/MASH pathogenesis and prognosis. Although some studies suggest comparable liver-related mortality between men and women, emerging evidence indicates that hormonal status, particularly menopause-associated estrogen deficiency, may significantly influence metabolic dysfunction and fibrosis progression in women (12).

## 2 Menstrual cycle and menopause

The menstrual cycle is regulated by the hypothalamic-pituitary-ovarian (HPO) axis and is characterized by coordinated hormonal fluctuations that ensure normal reproductive function (13). The cycle consists of the follicular phase, ovulation, and the luteal phase (13, 14). During the follicular phase, follicle-stimulating hormone (FSH) stimulates ovarian follicle development, resulting in progressively increasing estradiol concentrations (13, 14). Ovulation is triggered by a surge in luteinizing hormone (LH), while the luteal phase is characterized by increased progesterone production from the corpus luteum (13, 14). These cyclical hormonal changes are essential not only for fertility but also for maintaining metabolic homeostasis throughout the reproductive years (13, 15).

Beyond their reproductive role, estrogens exert important metabolic effects through regulation of glucose metabolism, insulin sensitivity, lipid homeostasis, adipose tissue distribution, mitochondrial function, and inflammatory signaling pathways (15, 16). Estrogen receptor signaling contributes to improved insulin sensitivity and regulation of hepatic lipid metabolism (16). Estrogen also promotes fatty acid oxidation and influences body fat distribution, favoring subcutaneous rather than visceral adipose tissue accumulation (15, 16). Consequently, premenopausal women generally exhibit a more favorable metabolic profile and a lower prevalence of metabolic syndrome and metabolic dysfunction-associated liver disease compared with age-matched men (15).

The menopausal transition represents a progressive decline in ovarian function accompanied by substantial hormonal changes (13, 17). During perimenopause, ovarian follicular reserve gradually diminishes, resulting in increasingly variable estrogen production and menstrual cycle irregularity (13, 18). Menopause is clinically defined as the permanent cessation of menstruation following twelve consecutive months of amenorrhea (17, 18). This transition is associated with persistently reduced estrogen and progesterone concentrations together with elevated follicle-stimulating hormone levels (13, 17). Menopause occurs as a consequence of ovarian follicular depletion and age-related reproductive endocrine changes (13, 18). These endocrine alterations extend beyond reproductive health and affect multiple physiological systems, including thermoregulation, sleep, cognition, emotional well-being, cardiovascular health, bone metabolism, and metabolic function (13, 17–19).

Increasing evidence suggests that menopause should be considered a systemic metabolic transition rather than solely a reproductive event (15, 20, 21). Estrogen deficiency contributes to visceral adiposity, impaired insulin sensitivity, dyslipidemia, and chronic low-grade inflammation, all of which are recognized drivers of metabolic dysfunction (20) (**Table 1**). Furthermore, postmenopausal women demonstrate increased accumulation of visceral and ectopic adipose tissue, including hepatic fat, compared with premenopausal women (20, 21). The interaction between hormonal decline, obesity, insulin resistance, and chronic inflammation provides the biological foundation for the menopause-obesity axis in MASLD and MASH (16, 21). Understanding physiological and metabolic changes occurring throughout the menopausal transition is therefore essential for elucidating the mechanisms underlying disease progression and for developing sex-specific preventive and therapeutic strategies (16, 20, 21).

**Table 1:** Hormonal and metabolic changes across the menopausal transition.

Stage	Estrogen levels	Body fat distribution	Insulin sensitivity	MASLD/MASH risk
Premenopause	High and cyclical	Predominantly subcutaneous	Higher	Lower
Perimenopause	Fluctuating	Increasing central adiposity	Declining	Moderate
Postmenopause	Persistently low	Predominantly visceral	Reduced	Higher

## 3 Methods for diagnosis of MASLD and MASH

Current diagnostic approaches for MASLD and MASH are broadly categorized into non-invasive and invasive modalities and generally include blood-based assessments, imaging techniques, and liver biopsy (22). Blood tests are commonly used to evaluate liver enzymes, metabolic abnormalities, lipid profiles, and inflammatory markers, although they often lack sensitivity and specificity for accurately staging disease severity (23). Imaging modalities have become increasingly important for non-invasive evaluation of hepatic steatosis and fibrosis, while liver biopsy remains the reference standard for confirming MASH and determining fibrosis stage despite its invasive nature and associated limitations (24).

### 3.1 Liver biopsy

Liver biopsy remains the reference standard for the diagnosis and staging of metabolic dysfunction-associated steatohepatitis (MASH), as it enables direct histological assessment of hepatic steatosis, hepatocyte ballooning, lobular inflammation, and fibrosis severity (22, 25). Histopathological evaluation allows differentiation between simple steatosis and steatohepatitis while providing important prognostic information regarding disease progression and fibrosis risk (22). In addition, liver biopsy facilitates fibrosis staging and assessment of disease activity, both of which are strongly associated with long-term clinical outcomes (26). However, despite its diagnostic value, routine liver biopsy is limited by its invasive nature, sampling variability, interobserver variability, procedure-related risks, patient discomfort, and cost (22). Consequently, substantial effort has focused on developing reliable non-invasive diagnostic methods capable of identifying steatosis, MASH, and fibrosis while reducing the need for biopsy (22).

### 3.2 Imaging techniques

Imaging modalities have significantly improved the non-invasive assessment of hepatic steatosis by enabling direct visualization and quantification of liver fat accumulation (24). Ultrasonography remains the most widely used first-line imaging technique due to its accessibility, low cost, and favorable safety profile (24). Although ultrasound performs reasonably well in detecting moderate-to-severe steatosis, its sensitivity is reduced in cases of mild hepatic fat accumulation and advanced fibrosis (27).

Computed tomography (CT) can also detect hepatic steatosis and provides detailed structural imaging; however, its clinical utility is limited by radiation exposure and relatively lower sensitivity for mild disease (28). In contrast, magnetic resonance imaging (MRI), particularly magnetic resonance imaging proton density fat fraction (MRI-PDFF), offers superior diagnostic accuracy for quantifying hepatic fat content across all stages of MASLD (29). MRI-PDFF has demonstrated excellent sensitivity and reproducibility and is increasingly utilized in both clinical research and therapeutic monitoring (30).

Controlled attenuation parameter (CAP), which is integrated with transient elastography, simultaneously evaluates hepatic steatosis and liver stiffness, thereby allowing combined assessment of steatosis and fibrosis severity (31). Quantitative MRI techniques are currently regarded as among the most accurate non-invasive methods for assessing hepatic steatosis, although their high cost and limited availability may restrict widespread use, particularly in resource-limited settings (32).

### 3.3 Blood-based biomarkers

Blood-based biomarkers represent an attractive non-invasive approach for the detection, monitoring, and risk stratification of metabolic dysfunction-associated steatotic liver disease (MASLD) and metabolic dysfunction-associated steatohepatitis (MASH) because they are widely available, relatively inexpensive, and easily integrated into routine clinical practice (33, 34). Standard laboratory assessment typically includes alanine aminotransferase (ALT), aspartate aminotransferase (AST), gamma-glutamyl transferase (GGT), lipid profiles, glucose metabolism parameters, and inflammatory markers, all of which may provide indirect evidence of hepatic injury and metabolic dysfunction (33, 34). However, individual biomarkers often demonstrate limited sensitivity and specificity, particularly for distinguishing simple steatosis from MASH or accurately assessing fibrosis severity (33, 34).

Recent advances have focused on the development of composite biomarker panels and novel serum markers designed to improve diagnostic performance and reduce reliance on invasive procedures (34, 35). These approaches integrate markers of hepatocellular injury, inflammation, extracellular matrix remodeling, and metabolic dysfunction to provide a more comprehensive assessment of disease activity (34, 35). Although several emerging biomarkers have shown promising diagnostic potential, further validation is required before widespread implementation in routine clinical practice (34, 35).

### 3.4 Non-invasive scoring systems

Several non-invasive scoring systems have been developed to facilitate the identification of hepatic steatosis and estimation of disease severity in patients with MASLD (33–36) (**Table 2**). Blood-based biomarker panels and scoring systems have emerged as practical non-invasive tools for identifying MASLD and estimating hepatic steatosis severity (34, 35). The Fatty Liver Index (FLI), which incorporates body mass index, waist circumference, triglycerides, and gamma-glutamyl transferase (GGT), is commonly used because of its simplicity and low cost, although its diagnostic performance for mild steatosis remains limited (34, 35).

**Table 2:** Commonly used non-invasive diagnostic scores in MASLD.

Score	Variables included	Primary purpose
FLI	BMI, waist circumference, triglycerides, GGT	Detection of hepatic steatosis
HSI	BMI, diabetes status, AST/ALT ratio	Identification of hepatic steatosis
SteatoTest	ALT, glucose, α2-macroglobulin, ApoA1, BMI	Estimation of liver fat accumulation
NAFLD Liver Fat Score	Fasting insulin, metabolic syndrome parameters, liver enzymes	Prediction of hepatic steatosis

The Hepatic Steatosis Index (HSI) combines body mass index, diabetes status, and the AST/ALT ratio and has demonstrated utility in biopsy-confirmed MASLD cohorts, although specificity may be suboptimal in some populations (33, 35). More complex biomarker models such as the SteatoTest incorporate multiple biochemical and clinical variables, including alanine aminotransferase, glucose, alpha-2 macroglobulin, apolipoprotein A1, and body mass index, to estimate liver fat accumulation (33). Similarly, the NAFLD Liver Fat Score integrates fasting insulin levels, metabolic syndrome parameters, and liver enzymes to improve diagnostic prediction; however, reliance on insulin measurements may limit routine clinical applicability (36).

Although these scoring systems provide accessible and cost-effective screening options, their ability to accurately stage steatosis and distinguish MASH from simple steatosis remains imperfect, highlighting the continued need for more reliable and standardized diagnostic biomarkers and multimodal diagnostic strategies (36).

Although these diagnostic approaches facilitate disease detection and risk stratification, obesity remains one of the principal drivers of disease progression from simple steatosis to MASH and fibrosis. Therefore, understanding the mechanisms linking obesity to hepatic injury is essential.

## 4 Obesity and MASH progression

### 4.1 Obesity-driven hepatic injury

The development of obesity-associated liver disease is driven by a complex interaction of metabolic, inflammatory, and endocrine abnormalities. A central mechanism underlying this process is insulin resistance, which disrupts normal glucose and lipid homeostasis and promotes excessive lipolysis, elevated circulating free fatty acids, and increased hepatic fat accumulation (37). Dysfunctional adipose tissue further contributes to disease progression through altered adipokine secretion, chronic low-grade inflammation, and local hypoxia, all of which are strongly implicated in the pathogenesis of MASLD and the progression to MASH. The burden of MASLD is particularly high among individuals with obesity, with reported prevalence rates ranging from 50% to 90% in this population (38–40). These metabolic alterations facilitate hepatic steatosis, lipotoxicity, and oxidative injury, ultimately activating inflammatory and fibrogenic pathways that promote progression toward MASH, fibrosis, and cirrhosis (32).

Lipotoxicity represents a major pathological feature of obesity-related liver injury. Excessive intracellular lipid accumulation impairs mitochondrial activity and enhances reactive oxygen species (ROS) production, leading to oxidative damage and hepatocyte dysfunction (39). Sustained oxidative stress contributes to inflammation, cellular apoptosis, and activation of pathways involved in fibrogenesis and hepatocarcinogenesis (40). Consequently, obesity has been identified as an independent risk factor for hepatocellular carcinoma (HCC) and HCC-related mortality (25). A large meta-analysis involving over 1.5 million individuals demonstrated that pre-existing obesity was associated with approximately a twofold increase in HCC mortality risk, particularly among men and Western populations (41). Importantly, most hepatic triglycerides in MASLD originate from adipose tissue-derived free fatty acids rather than direct dietary fat intake, further emphasizing the central contribution of adipose tissue dysfunction in disease pathogenesis (38).

Sex-related hormonal differences also play a significant role in MASLD susceptibility and progression. Although obesity prevalence is slightly higher among women globally, MASLD is more frequently diagnosed in men, highlighting the influence of sex hormones on metabolic regulation (21). Testosterone has been associated with increased MASLD risk, whereas estrogen appears to exert protective metabolic effects through estrogen receptor-α signaling pathways that improve insulin sensitivity and suppress hepatic lipogenesis (21). However, following menopause, declining estrogen levels diminish these protective effects, placing women at increased risk of progressive fibrosis and advanced liver disease (21). Collectively, these observations indicate that obesity-related liver disease is multifactorial and influenced by both metabolic and hormonal determinants (**Table 3**).

**Table 3:** Representative studies examining the relationship between menopause, obesity, and MASH progression.

Study	Design	Population	Main findings
Ntikoudi et al., 2024 (19)	Scoping review	Midlife women	Menopausal status was associated with a higher prevalence of MASLD and adverse metabolic profiles.
Milani et al., 2025 (21)	Narrative review	Postmenopausal women	Estrogen deficiency contributes to visceral adiposity, insulin resistance, and progression of steatosis and fibrosis.
Julián et al., 2023 (44)	Review article	Adults with obesity and MASLD	Abdominal obesity and metabolic dysfunction were identified as major drivers of disease progression and fibrosis development.
Nowak et al., 2025 (80)	Review article	Patients with obesity-related liver disease	Obesity promotes progression from simple steatosis to MASH through lipotoxicity, insulin resistance, and chronic inflammation.
Boulos et al., 2025 (66)	Review article	Individuals with MASLD	Adipose tissue dysfunction contributes to hepatic inflammation, metabolic dysregulation, and fibrosis progression.

The progression from simple steatosis to steatohepatitis and advanced fibrosis is influenced by the cumulative effect of multiple metabolic and environmental risk factors (42). Longitudinal studies have demonstrated that weight gain substantially accelerates disease progression by worsening insulin resistance and promoting hepatic fat deposition. In particular, gaining more than 5 kg has been associated with increased risk of fibrosis progression and worsening liver injury over time (37). Among obesity-related factors, visceral adiposity appears to be especially important in determining fibrosis risk and disease progression.

The coexistence of additional metabolic abnormalities further amplifies the risk of advanced liver disease. In a population-based study evaluating liver stiffness measurements, individuals with MASLD and dysglycemia demonstrated significantly higher rates of moderate-to-advanced fibrosis compared with the general population (43). Interestingly, premenopausal women appeared to have lower fibrosis risk, likely reflecting the protective metabolic effects of estrogen (43).

### 4.2 Role of diet and lifestyle

The pathogenesis of MASLD and its progression to MASH is multifactorial and results from complex interactions between genetic susceptibility, epigenetic modifications, metabolic dysfunction, environmental exposures, and lifestyle-related factors (44). Several biological mechanisms contribute to disease development, including dysregulated hepatic lipid metabolism, systemic insulin resistance, endocrine disturbances, cardiovascular comorbidities, chronic inflammation, and alterations in the gut-liver axis (45, 46). Emerging evidence also highlights the contribution of hormonal imbalance, immune-inflammatory activation, gut microbiota dysbiosis, environmental toxins, and circadian disruption to MASLD progression (47–49). Environmental and behavioral factors remain among the most important modifiable contributors to disease onset and progression (50).

Weight gain generally occurs because of chronic energy imbalance characterized by excessive caloric intake and insufficient energy expenditure (51). Sedentary behavior and low levels of physical activity further exacerbate metabolic dysfunction and adiposity (51). Diets rich in saturated fats, refined carbohydrates, fructose, and ultra-processed foods promote hepatic lipid accumulation, insulin resistance, and inflammatory activation, thereby accelerating steatosis and liver injury (30). In addition, environmental pollutants such as phthalates and bisphenols have increasingly been implicated in metabolic liver disease pathogenesis through endocrine-disrupting and inflammatory mechanisms (52, 53). Social determinants of health, occupational factors such as shift work, and unhealthy lifestyle patterns also contribute significantly to MASLD risk and progression (54, 55).

#### 4.2.1 Role of weight loss

Lifestyle modification and sustained weight reduction remain the cornerstone of MASH management and are currently considered first-line therapeutic strategies (56, 57). Weight loss has consistently demonstrated beneficial effects on hepatic steatosis, liver enzyme abnormalities, insulin resistance, and histopathological features of MASH (58). Clinical evidence supports a dose-dependent relationship between the magnitude of weight reduction and improvement in liver disease severity. A reduction of at least 5% of body weight has been associated with significant improvement in steatosis, whereas weight loss exceeding 7-10% is linked to MASH resolution and fibrosis regression (59, 60).

Caloric restriction independently improves hepatic inflammation, fibrosis, and metabolic parameters even in the absence of intensive physical activity (61). Moderate reductions in body weight achieved through energy restriction can significantly decrease liver fat content, waist circumference, serum aminotransferases, and cardiometabolic risk factors (62). In patients with severe obesity, prolonged energy-restricted diets may produce weight loss exceeding 10%, particularly when maintained for at least six weeks (63). Furthermore, combining caloric restriction with structured behavioral interventions and very-low-energy diets enhances long-term weight reduction outcomes compared with behavioral strategies alone (63, 64).

Sex-specific differences in metabolic response to weight loss have also been reported. Women may experience greater reductions in fat-free mass, hip circumference, and low-density lipoprotein cholesterol compared with men following intensive dietary interventions (65). Current international guidelines consistently recommend dietary caloric restriction combined with regular physical activity as the foundation of MASLD treatment (3, 66). Both aerobic exercise and resistance training have demonstrated efficacy in improving hepatic steatosis and metabolic health (67, 68).

#### 4.2.2 Role of dietary modifications

In addition to total caloric intake, dietary composition and eating behaviors significantly influence MASLD risk and progression. Western-style dietary patterns characterized by high intake of saturated fats, refined carbohydrates, sugar-sweetened beverages, and fructose are strongly associated with obesity, gut dysbiosis, systemic inflammation, and hepatic steatosis (30, 69). Even modest weight gain of 3–5 kg may increase the likelihood of developing MASLD (70). Meal frequency and snacking behaviors may also contribute to disease development, as frequent snacking has been independently associated with increased intrahepatic triglyceride accumulation (70).

The Western diet is additionally characterized by low consumption of fruits, vegetables, whole grains, fish, and antioxidant-rich foods, leading to deficiencies in dietary fiber, micronutrients, and anti-inflammatory compounds (71). In contrast, adherence to healthy dietary patterns such as the Mediterranean diet has been associated with lower MASLD prevalence and reduced fibrosis risk (59, 72–74). Diets rich in fruits, vegetables, olive oil, nuts, legumes, and fish provide antioxidant and anti-inflammatory benefits that may protect against hepatic injury (56). High adherence to Western dietary patterns has been linked to approximately double the risk of MASLD and greater fibrosis severity independent of body mass index and physical activity (70, 75). Experimental studies further support these observations, demonstrating that Western-style diets can induce steatosis, inflammation, and fibrosis through alterations in gut microbiota composition and metabolic signaling pathways (75).

Excessive intake of processed foods also contributes to high dietary sodium consumption, which has been associated with chronic metabolic inflammation and increased cardiovascular risk (76, 77). Ultra-processed foods may further exacerbate obesity, insulin resistance, and inflammatory activation involved in MASLD progression (78). Although the direct role of sodium intake in MASLD remains incompletely understood, its close association with hypertension and cardiovascular disease highlights the importance of dietary quality in reducing overall morbidity and mortality risk (79).

Overall, caloric restriction remains the most evidence-based intervention for improving MASLD outcomes. However, optimizing dietary quality through reduction of saturated fats, refined sugars, and fructose, while promoting Mediterranean-style dietary patterns and regular physical activity, appears essential for improving both hepatic and metabolic health.

**Figure 1:**
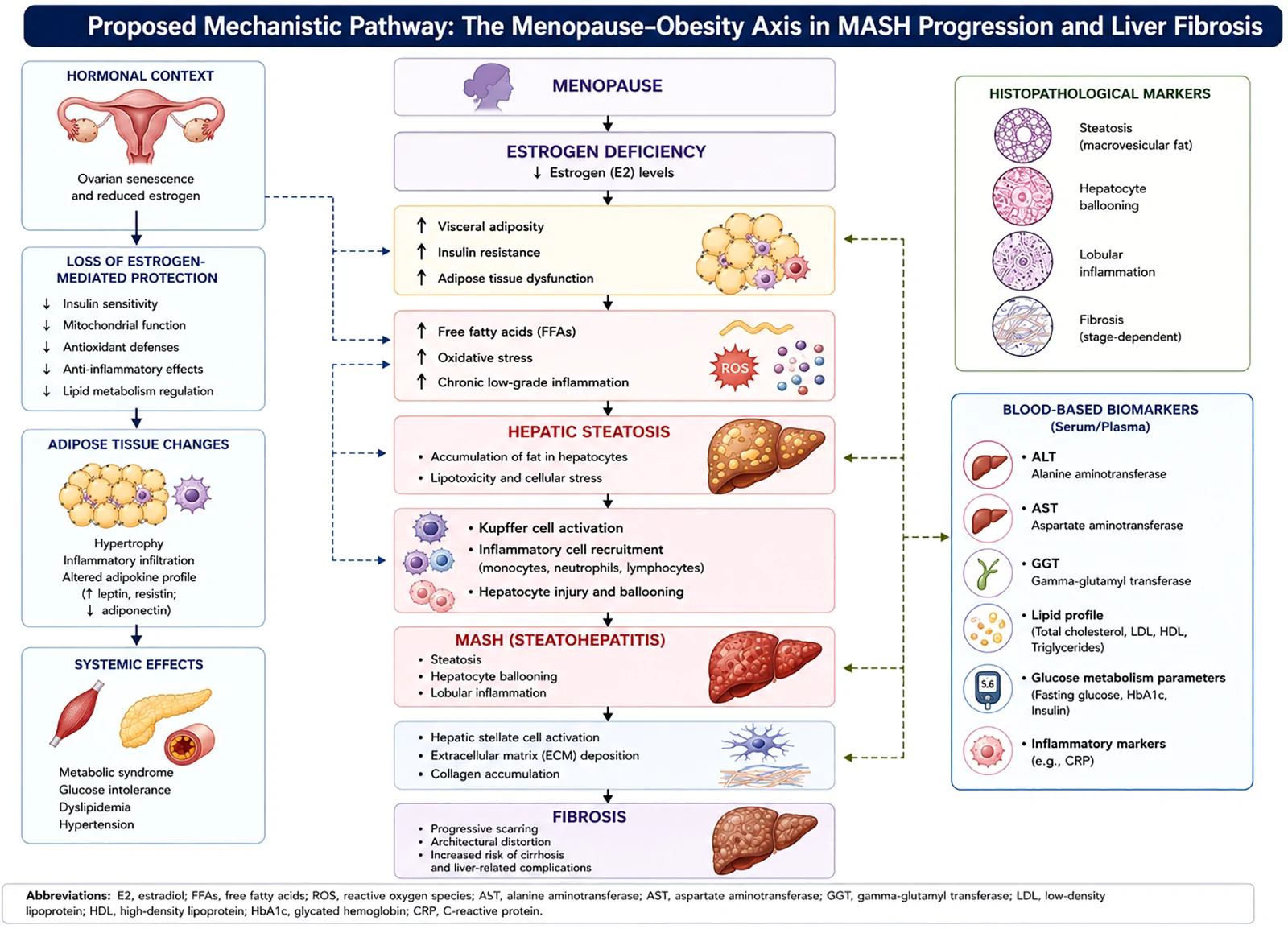
Proposed mechanistic pathway linking menopause, obesity, and MASH progression.

Estrogen deficiency during menopause promotes visceral adiposity, insulin resistance, and adipose tissue dysfunction, resulting in increased free fatty acid flux, oxidative stress, and chronic inflammation. These processes contribute to hepatic steatosis, hepatocellular injury, and progression to MASH and fibrosis. Histopathological markers, blood-based biomarkers, and risk scores relevant to disease assessment are also shown in **Figure 1**.

Importantly, the metabolic consequences of obesity may be further amplified by menopause-associated estrogen deficiency, creating a synergistic pathway that accelerates MASH progression in postmenopausal women.

## 5 Menopause-obesity axis in MASH

### 5.1 Estrogen decline and metabolic dysfunction

While obesity independently contributes to hepatic injury, menopause-associated estrogen deficiency may further amplify these metabolic disturbances and accelerate disease progression. Estrogen plays a central role in regulating hepatic lipid metabolism, insulin sensitivity, mitochondrial function, and adipose tissue distribution through estrogen receptor-mediated signaling pathways (21, 23). Consequently, the loss of estrogenic protection during menopause creates a metabolic environment that favors hepatic steatosis, inflammation, and fibrosis (21, 23).

One of the principal mechanisms linking menopause to MASH progression is the development of insulin resistance. Reduced estrogen levels impair glucose homeostasis and insulin signaling, resulting in increased hepatic glucose production, enhanced *de novo* lipogenesis, and excessive accumulation of lipids within hepatocytes (81). These alterations promote hepatic steatosis and increase susceptibility to lipotoxic injury, oxidative stress, and hepatocellular damage (66, 81).

Menopause is also associated with increased visceral adiposity and ectopic fat deposition. Compared with premenopausal women, postmenopausal women demonstrate greater accumulation of metabolically active visceral adipose tissue, which releases excessive amounts of free fatty acids, adipokines, and pro-inflammatory mediators into the portal circulation (21, 64, 79). This increased flux of free fatty acids to the liver promotes triglyceride accumulation, mitochondrial dysfunction, oxidative stress, and activation of inflammatory pathways involved in MASH development (21, 66, 80).

Beyond steatosis, estrogen deficiency may contribute directly to fibrosis progression. Chronic lipotoxicity, oxidative stress, and inflammatory signaling activate hepatic stellate cells, leading to extracellular matrix deposition and progressive fibrogenesis (21, 82–84). Experimental and clinical evidence suggests that estrogen exerts anti-inflammatory and anti-fibrotic effects; therefore, the loss of estrogen-mediated protection during menopause may facilitate progression from simple steatosis to steatohepatitis and advanced fibrosis (21, 23).

Collectively, estrogen deficiency amplifies obesity-related metabolic dysfunction through interconnected effects on insulin resistance, visceral adiposity, inflammation, and fibrogenesis. These mechanisms provide a biological explanation for the increased susceptibility of postmenopausal women to MASH progression and advanced liver fibrosis (21, 23).

### 5.2 Histopathological markers

The histopathological progression of MASLD encompasses a continuum ranging from simple steatosis to MASH, advanced fibrosis, and cirrhosis. Hepatic steatosis, characterized by excessive lipid accumulation within hepatocytes, represents the earliest and most common histological feature of the disease (25). Persistent metabolic dysfunction, insulin resistance, obesity-related lipid overload, and menopause-associated hormonal changes contribute to progression beyond isolated steatosis and promote hepatocellular injury and inflammatory activation (21, 80).

Hepatocyte ballooning is considered a hallmark histopathological feature distinguishing MASH from uncomplicated steatosis (84). Ballooned hepatocytes exhibit cellular enlargement, cytoskeletal disruption, and degeneration resulting from lipotoxic injury, oxidative stress, and mitochondrial dysfunction (85). These morphological changes reflect progressive hepatocellular damage and are closely associated with disease activity and fibrosis progression.

Chronic hepatic inflammation further drives disease severity in MASH (40). Histological examination frequently demonstrates inflammatory cell infiltration within the hepatic lobules, reflecting persistent activation of immune and inflammatory pathways (21, 86). Obesity-associated adipose tissue dysfunction and menopause-related estrogen deficiency contribute to increased cytokine production and sustained hepatic injury, thereby promoting progression toward steatohepatitis (16, 21, 66).

Fibrosis represents the most clinically significant histopathological determinant of prognosis in MASLD and MASH (26). Progressive extracellular matrix deposition and collagen accumulation result from activation of hepatic stellate cells in response to chronic inflammation, oxidative stress, and lipotoxicity (87). Estrogen deficiency may further contribute to fibrogenesis through impaired metabolic regulation and altered inflammatory signaling pathways, potentially explaining the increased risk of advanced fibrosis observed in postmenopausal women (16). Advanced fibrosis and cirrhosis are strongly associated with liver-related morbidity, mortality, and hepatocellular carcinoma risk, highlighting the importance of early disease identification and intervention (26, 87).

### 5.3 Biomarkers

The identification of reliable biomarkers for MASLD and MASH has become increasingly important for disease monitoring, fibrosis risk stratification, and the assessment of disease progression. While conventional laboratory biomarkers such as alanine aminotransferase (ALT), aspartate aminotransferase (AST), and gamma-glutamyl transferase (GGT) are widely used in clinical practice, their diagnostic accuracy for distinguishing simple steatosis from MASH and for assessing fibrosis severity remains limited when used individually (23, 33, 34).

Several emerging biomarkers have demonstrated potential utility in identifying patients at increased risk of disease progression and fibrosis. Cytokeratin-18 (CK-18) fragments, released during hepatocyte apoptosis, are among the most extensively studied biomarkers and have been associated with MASH activity and hepatocellular injury. Similarly, the N-terminal propeptide of type III collagen (PRO-C3), a marker of extracellular matrix formation and fibrogenesis, has shown promising performance in identifying patients with advanced fibrosis and active fibrotic remodeling. These biomarkers provide mechanistic insight into disease activity and may improve risk stratification beyond routine biochemical testing (33, 34).

Composite biomarker panels have also been developed to improve diagnostic accuracy. The Enhanced Liver Fibrosis (ELF) test combines serum markers of matrix turnover, including hyaluronic acid, tissue inhibitor of metalloproteinase-1 (TIMP-1), and procollagen III amino-terminal peptide (PIIINP), and has demonstrated utility for identifying advanced fibrosis in MASLD. Similarly, FibroMeter integrates biochemical and clinical parameters to estimate fibrosis severity and may assist in non-invasive disease assessment. Although these approaches show considerable promise, additional validation studies are required before their widespread implementation in routine clinical practice and menopause-specific populations (33, 34).

### 5.4 Immune response

Obesity and menopause synergistically promote chronic low-grade inflammation that contributes significantly to MASH progression and hepatic fibrosis. Estrogen deficiency is associated with increased production of pro-inflammatory cytokines and impaired regulation of immune and metabolic pathways, thereby creating a pro-inflammatory hepatic environment (16, 21). Simultaneously, dysfunctional visceral adipose tissue releases excessive amounts of free fatty acids, adipokines, cytokines, and chemokines that further promote hepatic inflammation and metabolic dysfunction (66, 80).

Within the liver, activation of resident macrophages (Kupffer cells), recruitment of inflammatory cells, oxidative stress, and lipotoxic injury contribute to hepatocellular damage and progression from simple steatosis to steatohepatitis (86, 88). Persistent inflammatory signaling stimulates hepatic stellate cell activation and extracellular matrix deposition, ultimately driving fibrosis development (87). The loss of estrogen-mediated anti-inflammatory effects following menopause may therefore represent a key mechanism linking obesity, metabolic dysfunction, and progressive liver injury in postmenopausal women (16, 21).

### 5.5 Risk scores and disease stratification

In the context of the menopause-obesity axis, non-invasive risk scores are increasingly used to identify individuals at increased risk of steatohepatitis and fibrosis and to facilitate disease stratification in clinical practice. Commonly used tools include the Fatty Liver Index (FLI), Hepatic Steatosis Index (HSI), SteatoTest, and NAFLD Liver Fat Score, which integrate anthropometric, metabolic, and biochemical variables associated with obesity and metabolic dysfunction (33–36). These scoring systems provide practical and cost-effective approaches for population screening and risk assessment, particularly in individuals with obesity-related metabolic abnormalities. Although they cannot replace histological evaluation, they may assist clinicians in identifying patients who require further investigation and closer monitoring. In postmenopausal women, where obesity, insulin resistance, and hormonal changes frequently coexist, such tools may be particularly valuable for early identification of individuals at increased risk of MASLD progression and fibrosis (33–36).

## 6 Therapies

### 6.1 Pharmacological therapies

The growing global burden of MASLD and MASH has accelerated the development of targeted pharmacologic therapies aimed at obesity, insulin resistance, inflammation, and fibrosis. Among the most promising agents are glucagon-like peptide-1 (GLP-1) receptor agonists, which have demonstrated substantial benefits in weight reduction, glycemic control, and hepatic steatosis improvement (88). Semaglutide and related GLP-1 agonists have shown significant reductions in liver fat content and improvement in metabolic dysfunction among patients with obesity-associated MASH (89). Beyond their metabolic effects, GLP-1 receptor agonists may also exert anti-inflammatory and hepatoprotective actions that contribute to slowing disease progression (90).

Tirzepatide, a dual glucose-dependent insulinotropic polypeptide (GIP) and GLP-1 receptor agonist, has recently emerged as a promising therapeutic strategy for obesity-associated MASLD. Clinical studies indicate that tirzepatide may produce substantial weight reduction while improving insulin resistance, hepatic steatosis, and cardiometabolic risk factors (91, 92). Its dual incretin activity appears to provide greater metabolic benefits compared with conventional GLP-1 receptor agonists, making it a particularly attractive option for patients with obesity-driven MASH.

Resmetirom, a selective thyroid hormone receptor-β agonist, represents one of the most important recent advances in MASH pharmacotherapy (78). By selectively targeting hepatic lipid metabolism, resmetirom reduces liver fat accumulation and improves steatohepatitis and fibrosis outcomes in clinical trials (100). Recent evidence suggests that thyroid hormone receptor agonists may effectively address both metabolic dysfunction and hepatic fibrogenesis, offering a disease-modifying therapeutic approach for MASH patients (78).

### 6.2 Hormone-based and emerging therapies

Hormone-based therapies have gained increasing attention in postmenopausal MASLD because of the recognized protective role of estrogen in hepatic and metabolic regulation (21).

Hormone replacement therapy (HRT) may improve insulin sensitivity, reduce visceral adiposity, and attenuate hepatic lipid accumulation through modulation of estrogen receptor signaling pathways (21). Experimental and clinical evidence also suggests that estrogen exerts anti-inflammatory and anti-fibrotic effects that may slow progression from steatosis to fibrosis in postmenopausal women (89). However, despite these potential benefits, the long-term safety and overall clinical utility of HRT in MASLD populations remain incompletely understood and require further investigation.

In parallel, several emerging anti-fibrotic therapies are under active investigation for advanced MASH. These therapeutic approaches target key pathways involved in hepatic inflammation, oxidative stress, stellate cell activation, and extracellular matrix deposition (92). Novel agents including fibroblast growth factor analogs, thyroid hormone receptor agonists, and metabolic pathway modulators may provide future opportunities for personalized treatment strategies aimed at preventing fibrosis progression and cirrhosis development (93).

## 7 Future perspectives

The growing recognition of sex-specific differences in MASLD and MASH highlights the need for more individualized and precision-based therapeutic approaches. Increasing evidence suggests that hormonal status, adipose tissue distribution, and metabolic responses differ substantially between men and women, particularly after menopause, influencing disease susceptibility, fibrosis progression, and treatment outcomes (21). Future research should therefore prioritize sex-specific medicine approaches that integrate hormonal, metabolic, and inflammatory pathways into disease stratification and therapeutic decision-making. In postmenopausal women, estrogen deficiency appears to accelerate visceral adiposity, insulin resistance, and hepatic fibrogenesis, emphasizing the importance of menopause-centered clinical frameworks in MASH management (21).

Another promising area involves the development of personalized biomarkers capable of improving early diagnosis, risk prediction, and monitoring of treatment response. Current non-invasive biomarkers often demonstrate limited sensitivity and specificity across heterogeneous patient populations. Emerging biomarker strategies incorporating metabolomics, lipidomics, inflammatory mediators, and genetic profiling may facilitate more precise identification of patients at high risk for progressive MASH and fibrosis (94). Integration of multi-omics technologies with imaging and clinical parameters may further enhance individualized risk assessment and therapeutic targeting.

Future clinical research should also focus on menopause-specific therapeutic interventions and dedicated clinical trials involving postmenopausal women with MASLD and MASH. Despite the recognized influence of estrogen deficiency on disease progression, women remain underrepresented in many MASLD clinical studies. Recent literature emphasizes the need for clinical trials specifically evaluating hormone-based therapies, metabolic interventions, and anti-fibrotic strategies in postmenopausal populations (95). Future individualized management may also benefit from integrating evidence regarding the magnitude of weight loss, dietary factors such as ultra-processed food consumption, incretin-based therapies, and current and emerging pharmacological regimens for MASH (96–99). Additionally, expanding research on sex-specific therapeutic responses may improve understanding of how obesity, menopause, and metabolic dysfunction interact to influence disease progression and treatment efficacy (92). Collectively, these advances may support the transition toward precision medicine and more effective individualized care in menopause-associated MASLD and MASH.
